# Petal senescence in cut roses

**DOI:** 10.1093/hr/uhaf342

**Published:** 2026-03-01

**Authors:** Saretta N Paramita, Denis Saint-Marcoux, Sylvie Baudino, Guillaume Beaugey, Sonja Meilland, Jean-Claude Caissard

**Affiliations:** Laboratoire de Biotechnologies Végétales appliquées aux Plantes Aromatiques et Médicinales UMR 5079, Université Jean Monnet Saint-Etienne, CNRS, F-42023 Saint-Etienne, France; Laboratoire de Biotechnologies Végétales appliquées aux Plantes Aromatiques et Médicinales UMR 5079, Université Jean Monnet Saint-Etienne, CNRS, F-42023 Saint-Etienne, France; Laboratoire de Biotechnologies Végétales appliquées aux Plantes Aromatiques et Médicinales UMR 5079, Université Jean Monnet Saint-Etienne, CNRS, F-42023 Saint-Etienne, France; Meilland International, F-83340 Le Luc en Provence, France; Meilland International, F-83340 Le Luc en Provence, France; Laboratoire de Biotechnologies Végétales appliquées aux Plantes Aromatiques et Médicinales UMR 5079, Université Jean Monnet Saint-Etienne, CNRS, F-42023 Saint-Etienne, France

## Abstract

Roses (*Rosa* sp.) are highly valued ornamental plants, with over 25 000 cultivars created by breeders, among which cut roses dominate the global flower market. Flowers of these cultivars can last up to 20 days in a vase from the moment they are cut, which is not the case for garden roses. This review examines whether the vase life of cut roses resembles or differs from natural flower senescence, focusing on the phytohormonal processes involved in both scenarios. We first compare petal senescence with other senescence phenomena and then examine genes related to hormone action. Finally, we show the similarities between senescence in cut roses and that of standing roses. We conclude that, despite the existence of similarities, including the involvement of ethylene in petal senescence, comparative studies between cut and uncut roses would be useful, both for basic research and to improve the selection of varieties with long vase life.

## Introduction

Roses (*Rosa* sp.) are among the most important horticultural plants and have been highly appreciated for their ornamental values since ancient times. From about 10 rose species, crossbreeding practices have led to more than 25 000 existing cultivars [1]. Rose varieties and cultivars can be classified into three main categories: species (wild roses and natural crossings), old garden roses (varieties existing before 1867), and modern roses (varieties existing after 1867, when the first tea hybrid rose, cultivar ‘La France’ was introduced). Modern roses can further be divided according to their uses: garden roses, potted roses, and cut roses [2, 3].

Cut roses represent the largest part of the world rose market, both in terms of volume and revenues. According to growers and bibliographic data [4, 5], areas such as China (19 420 ha), Kenya (3300 ha), Colombia (3000 ha), and Ecuador (3000 ha) are the main sources of production, while Ethiopia (900 ha), Europe (less than 500 ha), Japan (200 ha), and the USA (less than 30 ha) are lesser contributors. The Netherlands may not produce a large quantity of rose stems (less than 20 ha), but the country serves as the central hub for 60% of the global flower trade, with a particular emphasis on roses. Approximately 15 to 20 billion rose stems are sold each year, making roses one of the most significant cut flowers, second only to chrysanthemums. Of this total, roughly 70% are intended for domestic markets, in countries, such as Brazil, Mexico, Japan, Iran, South Africa, China, and India. The remaining 30% are exported, with Colombia, Ecuador, Kenya, Ethiopia, and India being the primary exporting countries. The USA stands as one of the largest importers, with roses mostly originating from Latin America, while Europe imports its roses primarily from East Africa and Latin America.

As a consequence of the production sites being dispatched worldwide, cut roses undergo a long series of procedures from the moment they are cut to reaching consumers' tables and being placed in a vase [6–10]. These include refrigeration, dehydration, storage, packaging, and transportation. Thus, flowers not yet fully opened are harvested early in the morning and subsequently placed in water-filled buckets under cold and dark conditions to put the flowers in a ‘dormant state’. From the cultivation site, flowers are then transported in 2 to 3 days to trading platforms, such as those in The Netherlands, either tightly packed in cardboard boxes in dry conditions, or in water-filled buckets for shorter trips. At auction sites, flowers undergo rehydration and cooling before being auctioned and sold to buyers. Wholesalers and flower shops can store the roses for up to three days before finally reselling and transporting them to the customer’s home.

Some breeding objectives are shared across all rose types, such as those related to flower color, flower shape, adaptability to the climate of the regions of production, pest control, and productivity [11–15]. However, in relation to the production and sale conditions described above, the selection process for cut roses also includes post-harvest qualities, such as vase life (VL), which is the time during which the rose flower keeps its ornamental qualities, mold control, and resilience of the flower to transport conditions [16–18]. As such, cut roses exhibit less phenotypical diversity particularly in terms of flower shape, fragrance, and VL. Flowers are often cupped, fully double, and typically unscented, although some fragrant cut roses can, on rare occasions, be found on the market today.

Any flower is a short-lived organ whose fate is to undergo profound transformation following pollination. During this process, part of the flower will die, such as petals. If no successful pollination occurs, the entire flower will eventually disappear. This process is called senescence and has been extensively studied. In this review, we sought to clarify whether the death of a cut rose can be compared to the natural process of senescence occurring on the plant. To this aim, we will first review the current knowledge of flower senescence, with a particular emphasis on roses. We will then detail the different mechanisms by which cut flowers die and examine to what extent the two processes could overlap.

### Flower senescence

**Figure 1:**
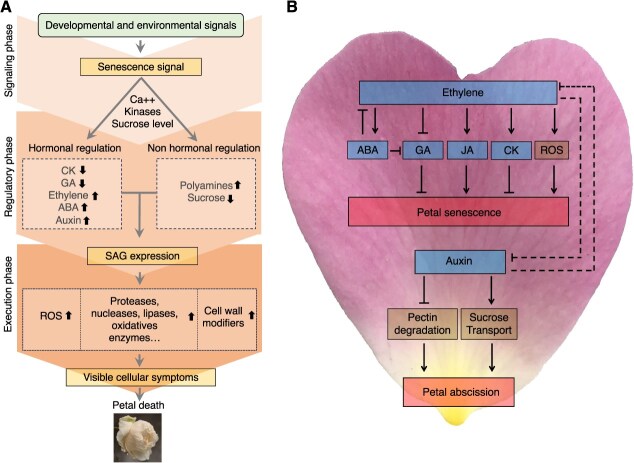
General overview of petal senescence and crosstalks in different signaling pathways (for a more detailed diagram see [19]). (A) Conceptual diagram of petal senescence in ethylene-sensitive flowers (adapted from [20] and [21]). (B) Current knowledge on signaling pathways in roses (adapted from [22] and [23]).

The word ‘senescence’ has been used in a scientific context for about a century. It comes from the Latin word *senescere* which means ‘to grow old’. In the field of plant biology, senescence was initially viewed as an inevitable and passive aging process. Today, it is widely accepted that senescence is an integral part of differentiation and development, involving coordinated signaling pathways (Fig. 1). Regarding flower development, it consists of several steps starting from flower growth and opening, until reaching its final stage, flower senescence which leads to the flower's death (Fig. 1A). Flower opening includes petal expansion and unfolding, anthesis, and petal senescence, which could be sometimes triggered by pollination, e.g. like in carnation [24]. A flower is composed of several organs, each of which can undergo senescence phenomena, as, e.g. in Arabidopsis [22, 25, 26]. However, horticulturists and scientific publications consider petal senescence as the most important phenomenon of floral senescence, and very often use the terms ‘petal senescence’ and ‘floral senescence’ as synonyms for cut flowers. In this paragraph we have therefore focused on petal senescence.

Petal senescence contributes to the remobilization of nutrients to other organs in the plant, such as developing seeds and young organs [27]. In addition, maintaining petals imposes an energetic cost on the plant since it consumes respiratory energy, nutrients, and causes water loss [28]. Therefore, it seems beneficial for plants to start petal senescence when petals are not needed anymore, in order to optimize fruit development and complete their reproduction. Nevertheless, flowers are also important aesthetic objects for human, and petal longevity determines the post-harvest quality and garden performance of ornamental plants. Delaying petal senescence is therefore of interest, and numerous research efforts have been dedicated to understanding the biochemical and physiological aspects of petal senescence.

Phenotypically, petal senescence is divided into two major groups, i.e. petal wilting and withering on the one hand and petal abscission on the other hand [29, 30]. Petal wilting is defined as petals losing their turgor while still attached to the flower and is often associated with petal withering linked with discoloration and slow desiccation of petals. In this way, recent articles reporting functional characterization of genes use the term petal senescence to address all types of these visible phenotypes [31, 32]. Indeed, the mechanisms underlying petal wilting, petal withering and petal discoloration appear to be concomitant [33]. Conversely, petal abscission describes the fall of still relatively turgid petals without wilting. In roses, it is noticeable that some varieties have petal wilting while others have petal abscission. Therefore, when the phenotype is clear, the term petal abscission is distinguished from petal senescence [34].

At the cellular level, characteristics of petal senescence include disruptions in cellular structure, increase in membrane permeability, and degradation of organelles, which can begin at early stages of flower development (Fig. 1A). The changes in the morphology and ultrastructure of the cells are accompanied by the remobilization of nutrients, decreased nucleic acid and protein content, increased nuclear condensation, DNA fragmentation, increased mRNA abundance, as well as increased activities of proteases, nucleases, and lipases [35–42]. Processes associated with petal senescence often start before the first visible signs of petal deterioration become apparent [43]. For example, in cut iris (*Iris* x *hollandica*) petals, the closure of plasmodesmata begins before flower opening, blocking the transfer of sugars, phytohormones, and RNA molecules between neighboring cells [36]. This is followed by a decrease in the number of small vacuoles, an increase in vacuolar size, as well as the degradation of cytoplasm and most organelles, such as endoplasmic reticulum and ribosomes. In cut Peruvian lily (*Alstroemeria*) flowers, the increase in protease activity and the decrease in lipid content occurs gradually, starting as early as before flower opening. Other features, such as protein degradation, loss of membrane integrity, and DNA fragmentation, accelerate when the first indications of petal deterioration become evident [37]. Similar events have also been reported in petunia (*Petunia* x *hybrida*) [38, 39], cut carnation (*Dianthus caryophyllus*) [35], and cut lily (*Lilium longiflorum*) flowers [42]. In petunia, petal senescence is also accompanied by nutrient remobilization, which involves the transfer of different nutrients from petals depending on whether the flower has been pollinated or not [41]. Additionally, it has been reported that the collapse of the vacuolar membrane (tonoplast), which is considered as the actual moment of cellular death, takes place before the collapse of the plasma membrane [33]. Each of these senescence-associated events occur at various moments during flower development, but the precise factor that initiates the actual death of the flower remains uncertain.

Petal senescence can also be categorized based on its biochemical regulation (Fig. 1B). This classification distinguishes between senescence initiated by ethylene (in ethylene-sensitive flowers), and senescence initiated by other plant hormones independently of ethylene (in ethylene-insensitive flowers) [29, 44]. Ethylene-sensitive flowers are characterized by a peak in ethylene biosynthesis, often triggered by events, such as pollination and fertilization. This surge in ethylene production induces an autocatalytic process, where the produced ethylene acts as a catalyst for its own synthesis. Subsequently, ethylene activates key enzymes within petal cells, leading to the breakdown of major macromolecules and the degradation of cellular organelles, ultimately initiating petal senescence [22]. It is considered that it is equivalent to programmed cell death (PCD) [19]. Among major ornamental species, the likes of carnation, rose, petunia, and orchid (*Phalaenopsis*) fall into the category of ethylene-sensitive flowers. Indeed, roses are generally considered as ethylene-sensitive flowers [44], although different rose varieties may display varying degrees of responsiveness to ethylene [45, 46]. By contrast, ethylene-insensitive flowers rely on other phytohormones as the primary regulators of senescence, with ethylene playing a minor role or having minimal impact.

### Petal senescence versus programmed cell death

Senescence is frequently associated with other terms related to cell death, including PCD, apoptosis, autophagy, and necrosis. The precise definition of senescence remains a complex subject of discussion. A cell is considered dead either when it has lost the integrity of its plasma membrane, when the cell has undergone complete fragmentation into discrete bodies (often referred to as apoptotic bodies), or when its corpse/fragments have been engulfed by an adjacent cell *in vivo* [47]. The term PCD does not refer to death itself, but rather refers to the genetic processes leading to the moment of death and degradation [48]. In animals, PCD is distinguished into three major classes based on their morphological features: apoptosis, autophagy, and necrosis [20, 22, 29, 47] (Table 1).

**Table 1:** Ultrastructural criteria defining PCD in animals and plants

**PCD class**	**Defining criteria**	**Other criteria**
Animals [47]		
Apoptosis	Apoptotic bodies, or blebs on cell surface. Degradation in other cells after phagocytosis.	Chromatin condensation, nuclear fragmentation.
Autophagy	Increased numbers of autophagosomes, autolysosomes, and small lytic vacuoles.	
Necrosis	None of the above defining criteria.	Cell swelling, organelle swelling, plasma membrane rupture.
Plants [49]		
Apoptosis-like PCD	Quick disruption of nucleus with nuclear shrinkage, chromatin condensation, nuclear fragmentation, and DNA laddering.	
PCD during leaf senescence	Slow cell death with thorough degradation of cell contents occurring before disruption of nucleus and vacuole.	
Vacuole-mediated PCD	Vacuolar swelling and collapse followed by degradation of cell contents (tonoplast and plasma membrane).	
Plants [50]		
Autolytic PCD	Rapid clearance of cytoplasm after rupture of tonoplast.	Chromatin condensation, increase in vacuolar volume (decrease in cytoplasm volume).
Non-autolytic PCD	No rapid clearance of cytoplasm (but can include tonoplast rupture).	Organelle swelling, no increase in vacuolar volume.

However, the classification of PCD in plants is still highly debated, as there is no direct equivalent of animal apoptosis [51]. Nevertheless, a classification which takes into account the presence of animal apoptotic-like characteristics in plants, such as nuclear shrinkage, chromatin condensation, nuclear fragmentation, and DNA laddering, was introduced by Fukuda *et al*. [49] (Table 1). This author divided plant PCD into three distinct groups: apoptosis-like PCD, cell death occurring during leaf senescence, and vacuole-mediated cell death. The identification of features in plants that are comparable to those observed in animal apoptosis, has led to the conclusion that apoptosis-like PCD does occur in plants, as argued in Dickman *et al*. [51]. However, Minina *et al*. [52] reported that apoptosis is not conserved between animals and plants. By contrast, van Doorn [50] avoided to rely on animal apoptotic features and proposed to classify plant PCD into two separate groups, autolytic and non-autolytic PCD (Table 1). Autolytic PCD was then defined as the rapid clearance of cytoplasm following the tonoplast rupture and primarily occurring during normal plant development and mild abiotic stress. Some research may also refer to autolytic PCD as vacuolar cell death [53]. Conversely, non-autolytic PCD was characterized as either the absence of rapid cytoplasmic clearance, or situations when such clearance occurs after the cell has already died. This form of PCD is primarily found during interactions between plants and pathogens. The complexity of PCD features in plants accounts for the ongoing uncertainties surrounding PCD classification, which can sometimes lead to confusion. For instance, when discussing petal senescence, some studies describe it as apoptosis-like PCD [54], while others categorize it as autolytic PCD [55, 56].

Furthermore, the terms PCD and senescence have also been a source of confusions which is well addressed in Van Doorn and Woltering [48]. Both terms refer to controlled processes associated with cell death. However, there are several key points that have been discussed to distinguish senescence from PCD. These include the reversibility of the processes and the scale or localization of the processes, i.e. whether they occur in individual cells, tissues, or organs. One suggestion to distinguish between senescence and PCD is the concept of reversibility, where senescence is considered a reversible process while PCD is seen as irreversible. However, this interpretation has also contributed to some confusion within the scientific community. Some scientists define senescence as the process that occurs after the point of no return, while others define it as the process that occurs before reaching the point of no return [48, 57]. All of these points have led to the confusion whether the terms PCD and senescence can be considered as similar events, distinct events or overlapping events. Nevertheless, in flowers, Rogers [43] mentioned that such distinction is unnecessary since flower deterioration is an irreversible programmed process leading to cell death. In line with works in this field [33, 43], both terms will not be distinguished when addressing flower senescence, particularly petal senescence, and will be used interchangeably in this review. However, PCD can be used when referring to the death of individual cells, while senescence is used to describe processes affecting tissues or whole organs. Finally, it is noticeable that horticulturists use senescence only for the visual aspect of the flower, not for the cellular or molecular mechanisms.

### Genes involved in physiological phenomena related to rose petal senescence

The literature on regulatory mechanisms of petal senescence has been consistently updated and enriched over time by various authors and will not be detailed here. For in-depth information on the molecular and physiological aspects of petal senescence, comprehensive reviews are available [19, 22, 30, 33, 56, 58]. In summary, petal senescence involves the expression of specific transcription factors (TFs) and genes encoding enzymes involved in various cellular processes. They concern cell wall degradation (involving enzymes such as β-galactosidases), protein synthesis and degradation (including enzymes, such as cysteine proteases, ubiquitin ligases, and metacaspases), nucleic acid synthesis and degradation (involving nucleases), lipid metabolism, β-oxidation, ethylene synthesis, perception and signaling, regulation of other hormones and crosstalk between them, sugar metabolism and transport (including invertases), and events related to oxidative stress and the production of reactive oxygen species (ROS), involving enzymes, such as catalases, superoxide dismutases, ascorbate peroxidases, and glutathione reductases. Some genes are upregulated while others are downregulated at different stages of petal development. These dynamic changes in gene expression reflect the complex, yet well-coordinated mechanisms of senescence, sometimes different from one genotype to another. Here, we will focus on three aspects of the regulation of senescence in rose petals: early transcriptional regulation by senescence-associated genes (SAGs), the implication of ROS, and the regulation by hormones (Fig. 1B). These mechanisms include petal senescence (Table S1) but also petal abscission (Table S2).

#### Senescence-associated genes

Significant research efforts on the regulatory mechanisms governing petal senescence have resulted in the identification of numerous senescence-related genes, including SAGs that are expressed early. While SAGs might refer to genes linked to senescence processes in a general sense, they are originally defined as genes having increasing expression levels during natural senescence [59, 60]. In addition, Lohman *et al*. [59] introduced the term ‘senescence-downregulated genes’ to describe genes that display downregulation during senescence, distinguishing them from SAGs. However, more recent papers make distinction neither between upregulated or downregulated genes, nor between natural or stress-induced senescence [20, 61].

Several genes directly involved in senescence processes have been identified and can serve as markers for senescence-associated events. One commonly used marker is *SAG12*, which encodes a cysteine protease and exhibits expression only in senescent tissues [59, 62]. It has been used several times as a marker of the senescence of rose petals (Table S1).

#### Reactive oxygen species

Recent findings demonstrated the implication of ROS in rose petal senescence (Table S1). ROS, including free radicals and hydrogen peroxide (H_2_O_2_), are produced during normal metabolism or are induced by stress. At night, *RhPIF8* (*PHYTOCHROME-INTERACTING FACTOR*) activates the expression of *RhBBX28 (B-BOX 28)*. *RhBBX28* represses the expression of *RhSDH1 (SUCCINATE DEHYDROGENASE 1*), which controls the dark-induced H_2_O_2_ accumulation and leads to petal senescence acceleration. The RhPIF8–RhBBX28 module is thus a critical player that controls flower senescence by governing mitochondrial ROS homeostasis in rose [31]. Furthermore, dehydration and aging increase ROS levels and result in precocious or accelerated senescence [63]. ROS induce *RhWRKY33a* (*WRKY domain 33a*) expression, which in turn upregulates directly the expression of *RhPLATZ9 (PLANT AT-RICH SEQUENCE AND ZINC-BINDING 9)*. *RhWRKY33* expression is also positively regulated by ethylene [64]. *RhPLATZ9* represses the expression of an apoplastic NADPH oxidase gene, *RhRbohD (RESPIRATORY BURST OXIDASE HOMOLOG)*, thus preventing excess ROS accumulation in cells [63]. Overexpression or silencing of *RhPLATZ9* and *RhWRKY33a* delays or accelerates flower senescence, respectively. Altogether, the *RhWRKY33a–RhPLATZ9–RhRbohD* module maintains ROS homeostasis to delay precocious senescence induced by dehydration and aging [63]. Moreover, it was recently shown that histone deacetylation by RhHDA15 (HISTONE DEACETYLASE 15) plays a crucial role in controlling rose petal senescence by fine-tuning ROS homeostasis [65]. The link between ethylene signaling and ROS production was also recently studied [66]. Ethylene reduces ascorbic acid production, leading to the accumulation of ROS and hastening petal senescence. A key player in this process is the TF RhHB22 (HOMEODOMAIN LEUCINE ZIPPER II 22), which inhibits the transcription of *RhGGP1 (GDP-I-GALACTOSE PHOSPHORYLASE 1)*, the rate-controlling enzyme in ascorbic acid biosynthesis.

#### Plant hormones

Plant hormones play an important role in the process of petal senescence, and they have been extensively studied in major ornamental flowers, well detailed in Ma *et al*. [22].

Ethylene is a primary hormone involved in senescence. It has been shown to accelerate petal senescence in many ethylene-sensitive flowers, and is also involved in regulating petal senescence in ethylene-insensitive flowers through interactions with other phytohormones. The biosynthesis and signaling pathways of ethylene have been studied in many species and are well documented [19, 22, 67–71]. Ethylene is perceived by a family of five membrane-bound receptors: ETR1, ETR2, ERS1, ERS2, and EIN4 (ETHYLENE RESPONSE 1 and 2, ETHYLENE RESPONSE SENSOR 1 and 2, and ETHYLENE INSENSITIVE 4)*.* In the absence of ethylene, the receptors keep their downstream component CTR1 (CONSTITUTIVE TRIPLE RESPONSE 1) active, resulting in the phosphorylation of EIN2, a multihelix membrane protein with a cytosolic domain that can serve as a TF. In the presence of ethylene, the receptors are inhibited and CTR1 becomes inactive, leading to less phosphorylation of EIN2 and trafficking of EIN2 to the nucleus. EIN2 plays a major role in ethylene sensitivity, and acts as a positive regulator of the ethylene signaling pathway. In the nucleus, EIN2 activates a family of TFs including EIN3, EIL1, and EIL2 (EIN3-LIKE 1 and 2), which in turn activate other TFs, such as ERF1 (ETHYLENE RESPONSE FACTOR 1). These TFs act as either activators or repressors of downstream ethylene-responsive genes. In roses, ethylene treatment induces gene-specific expression of several ethylene biosynthetic genes encoding different ACSs (1-AMINOCYCLOPROPANE-1-CARBOXYLATE SYNTHASE) (Table S1). *RhACS1* is involved in petal senescence and wounding, *RhACS3* in flower opening, ethylene dependency and wounding-inhibition, and only *RhACS2* is specific to senescence [46]. Moreover, the expression of *RhACS2* is rapidly induced by ethylene derived from gynoecia [72]. Ethylene treatment also results in a significant change in the expression of *RhACO1 (1-AMINOCYCLOPROPANE-1-CARBOXYLIC ACID OXIDASE 1)* [73].

Abscisic acid (ABA) is another phytohormone promoting petal senescence, whose action is closely related to ethylene in some rose varieties [74] (Table S1). Mayak and Halevy [75] reported increased endogenous ABA levels following ethylene treatment, in turn reducing ethylene production, possibly through a feedback mechanism. Moreover, 1-methylcyclopropene, an ethylene inhibitor, is able to inhibit ABA-induced flower senescence [76]. Ethylene induces the expression of *RhERF3* which activates the expression of an ABA biosynthesis gene, *RhNCED1 (9-CIS-EPOXYCAROTENOID DIOXYGENASE 1)*, by directly binding to its promoter, which accelerates petal senescence [77]. Interestingly, ABA also appear to be involved in delaying dehydration-related petal senescence. ABA modulates the expression of *RhABF2 (ABA-RESPONSIVE ELEMENT-BINDING FACTOR 2)*, a TF involved in ABA signaling pathway. *RhABF2* directly activates *RhFer1* (*FERRITIN 1*) involved in the maintenance of iron homeostasis affecting petal senescence [78].

Cytokinins (CKs) play a key role in regulating plant growth and development, and are generally considered to delay petal senescence in many species through several physiological processes, including inhibition of ethylene and ROS production, maintenance of membrane permeability, regulation of water balance, and deceleration of protein and nucleic acid metabolism [22]. In roses, CK level in petals is negatively correlated with flower senescence (Table S1). Ethylene-induced *RhHB6* (*HOMEODOMAIN LEUCINE ZIPPER I 6*) expression results in the upregulation of *RhPR10.1* (*PATHOGENESIS-RELATED PROTEIN 10.1*) expression and promotes CK production. Silencing of *RhPR10.1* results in the downregulation of CK signaling pathway genes *RhRR3*, *RhRR8*, and *RhRR9* (*RESPONSE REGULATORS 3, 8* and *9*) and the decrease in CK content, leading to accelerated petal senescence [79]. Silencing of *RhERF113* is followed by decreasing transcript accumulation related to CK biosynthesis and signaling, including *RhPR10.1, RhIPT5*, *RhIPT8*, *RhHK2*, *RhHK3*, *RhCRR3*, *RhCRR5*, *RhCRR8*, and *RhHB6* (*ISOPENTENYL TRANSFERASE 5* and *8, HISTIDINE KINASE 2* and *3, CYTOKININ RESPONSE REGULATOR 5* and *8*, and *HOMEOBOX PROTEIN 6)*. This silencing reduces CK content and accelerates petal senescence. Conversely, in non-silenced plants, RhERF113 delays ethylene-induced petal senescence by increasing CK content [80]. CKs were also found to be involved in petal abscission in roses [34] (Table S2). Indeed, during flower opening, CK content increases and induces the expression of *RhLOL1* (*LESION SIMULATING DISEASE 1-LIKE 1*) TF. *RhLOL1* interacts with another TF, *RhILR3* (*INDOLE-3-ACETIC ACID-LEUCINE RESISTANT 3),* leading to the activation of several *Aux/IAA* genes (*AUXIN/INDOLE-3-ACETIC ACID*), especially *RhIAA4–*1. This crosstalk between CK and auxin pathways accelerates petal abscission.

The role of auxin in petal senescence is still unclear but in roses, some reports have shown its implication in petal abscission by cell wall hydrolysis and cell growth at the base of the petal (Table S2). Downregulation of *RhIAA16 (INDOLE-3-ACETIC ACID 16)* resulted in precocious petal abscission, suggesting an important role of this gene in the phenomena [81]. Liang *et al*. [82] reported that auxin controls sucrose transport during petal abscission. An auxin signaling protein, RhARF7 (AUXIN RESPONSE FACTOR 7), regulates the expression of *RhSUC2* (*SUCROSE TRANSPORTER 2*), and together they repress petal abscission. As rose flowers age, *RhARF7* expression decreases, resulting in the reduced expression of *RhSUC2* expression and lower sucrose transport. Furthermore, the interaction between auxin and ethylene was found to regulate pectin degradation which is related with petal abscission. Ethylene inhibits the expression of *RhERF1*, while auxin induces *RhERF4* expression, and together, they repress the expression of *RhBGLA1 (β-GALACTOSIDASE 1)*. Reduced expression of *RhBGLA1* decreases pectin degradation and delays petal abscission [83]. A cell wall remodeling also occurs in the abscission zone through xyloglucan hydrolysis by XTH1 (XYLOGLUCAN ENDOTRANSGLUCOSYLASE/HYDROLASE 1) and protein destruction by CP1 (CYSTEINE PROTEASE 1) [84, 85].

Jasmonic acid (JA) also seems to be involved in the regulation of rose petal senescence, particularly by interacting with the ethylene signaling pathway (Table S1). JA, together with ethylene, induces the expression of *RhMYB108* (R2R3-MYELOBLASTOME TRANSCRIPTION FACTOR 108) TF, which in turn activates the expression of several *SAGs* and *NACs (NO APICAL MERISTEM, ARABIDOPSIS TRANSCRIPTION ACTIVATION FACTOR, CUP-SHAPED COTYLEDON)*, including *RhNAC029*, *RhNAC053*, *RhNAC092*, *RhSAG12* and *RhSAG113*, and promotes rose petal senescence [86]. Ca^2+^ concentration also plays a role through RhCBL4 (CALCINEURIN B-LIKE 4), a calcium receptor, which is induced by ethylene signaling [87]. Furthermore, the interaction between Ca^2+^-RhCBL4 and RhCIPK3 (CALCINEURIN B-LIKE PROTEIN INTERACTING PROTEIN KINASE 3) regulates the petal senescence by promoting the degradation of RhJAZ5 (JA RESPONSE REPRESSOR, JA ZINC FINGER INFLORESCENCE MERISTEM-DOMAIN) after phosphorylation [87]. This interaction removes the inhibition of senescence by RhJAZ5. Ethylene was also reported to induce petal senescence through RhCIPK6 and possibly RhCBL3 [32].

Gibberellic acid (GA) is a phytohormone, which also delays petal senescence (Table S1). Ethylene and ABA induce the expression of *RhHB1* (*HOMEODOMAIN LEUCINE ZIPPER I 1*), which directly binds to the promoter of a GA biosynthetic gene, *RhGA20ox1 (GIBBERELLIN 20-OXIDASE 1)*, and represses its expression. Reduced expression of *RhGA20ox1* results in decreased GA and promotes petal senescence [23]. Besides, GA signaling is reduced by ethylene through a second pathway decreasing [21]. Ethylene induces the expression of *RhSAF*, (*ETHYLENE-INDUCED F-BOX PROTEIN)*. RhSAF assembles with the GA receptor RhGID1, (GIBBERELLIN INSENSITIVE DWARF 1) which results in the ubiquitination and destruction of RhGID1, thus decreasing GA signaling.

In summary, ethylene, JA and ABA act as positive regulators of petal senescence, while CK and GA display antagonistic roles and delay petal senescence. Auxin may have both positive and negative effects on petal senescence, but it promotes petal abscission in some cultivars. It is worth noting that these complex processes happen sequentially during the development of the flower, from blooming to senescence or abscission, with, e.g. reduction in the transport of sucrose, modification of the antagonistic balances between hormones, and disruption of the homeostasis of different compounds [30, 88, 89].

### Is the VL of cut roses linked to petal senescence?

The VL of cut flowers, also known as longevity, shelf life, display life, keeping quality or lasting quality, is defined by the period starting from the placement of stems in a vase solution to the loss of visible ornamental value [90]. It is an important measure demonstrating the post-harvest quality of cut flowers after refrigeration, transport and rehydration. During these post-harvest procedures, cut rose flowers undergo a sequence of steps where they are first transported without water and subsequently placed in water, involving both dehydration and rehydration phases. During this process, some genes involved in ethylene perception and transduction have been characterized. The rehydration procedure induces the expression of *RhMKK9* (*MITOGEN-ACTIVATED PROTEIN KINASE KINASE 9*) which in turn activates the expression of the *RhMPK6-ACS1* module (*MITOGEN-ACTIVATED PROTEIN KINASE 6* – *ACS1*) in the gynoecia, leading to an ethylene burst promoting flower opening and petal senescence [91–93]. In the ethylene signaling pathway, the expression of *RhETR1*, *RhETR2*, *RhETR3*, *RhETR4*, contributes to the variation in ethylene sensitivity among different rose varieties [74, 94] and *RhETR3* appears to be the major regulator of flower opening and senescence in roses [91, 94, 95]. In addition, the expression of *RhCTR1* also increases during petal senescence [96]. Moreover, ethylene regulates both petal senescence and VL by interfering with other phytohormones, and with relative humidity and stomatal functioning [97].

**Figure 2:**
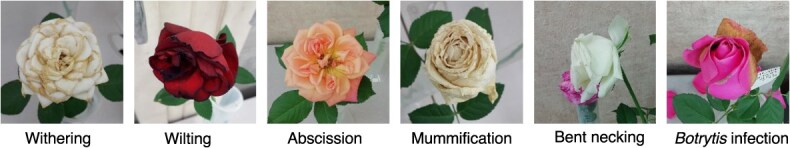
Vase life terminating symptoms in cut roses. The roses shown here are not commercial varieties. These are examples of photos taken during the selection process in Meilland greenhouse.

In laboratories, VL is evaluated using several different procedures. In general, these protocols begin with cutting the flowers in the morning, followed by storage procedures designed to mimic conditions within the supply chain. Subsequently, the stems are recut to a specific length, and the flowers are placed in a water-filled cylinder tube in controlled room conditions until the point at which a loss of visible ornamental value is observed. These protocols are generally designed based on the guidelines and standard protocols created by the Association of Dutch Flower Auctions named VBN (‘Vereniging van Bloemenveilingen in Nederland’)*.* Some studies also provided comprehensive details on the procedures, offering a standardized protocol for use in multiple laboratories worldwide [98, 99], even if some differences exist in the literature, particularly on senescence phenotypes, that could be really different form one rose to another (Fig. 2). However, these phenotypes seem to be all related to the water uptake of the flowers.

**Figure 3:**
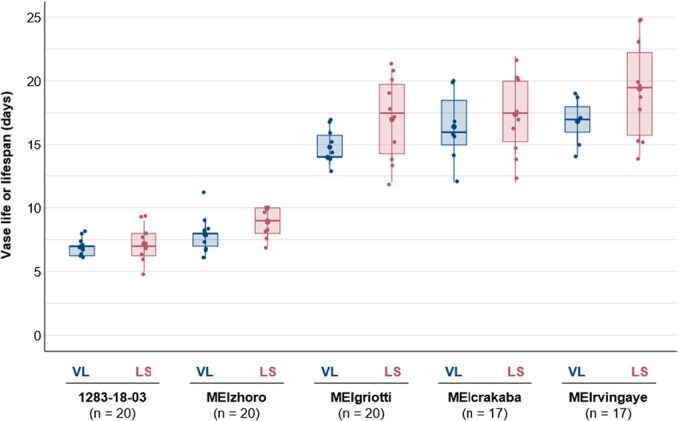
Comparison between the VL of cut roses and the LS of intact roses cultivated in pots according to the protocol of [88, 96, 97]. Briefly, roses were cut just before full-blooming stage for VL, or left standing in pots in Meilland's greenhouse for LS. This was considered as Day 0. Cut roses were put in 250 mL of tap water refilled each day. The number of days on the y-axis correspond to number of days between Day 0 and terminating symptoms of Fig. 2, except *Botrytis* infection that was excluded. Two-way ANOVA with interaction effects was done in the five cultivars (#1283-18-03, MEIzhoro, MEIgriotti, MEIcrakaba, MEIrvingaye) resulted in no significant difference between VL and LS values (*P* = 0.5843). *n*, number of analyzed samples.

Withering defines desiccated petal edges when petals turn brown or black on the edges, while wilting refers to petals that lose their turgor while still attached to the flower. Petal withering, wilting and abscission are the major VL terminating symptoms. It is tempting to relate withering and wilting to petal senescence because it is easy to observe that they look like dead flowers still attached to the plant. On the contrary, petal abscission describes the fall of petals while still relatively turgid, without wilting nor withering. Both phenomena, withering, wilting and abscission, can easily be observed in rose gardens.

Mummified flowers seem to be more related to defects in flower opening. In this case, flowers are unable to bloom and stay at an unopened stage while desiccating slowly. It is important to note that mummification generally leads to a longer VL evaluation since the symptoms are less visible and straightforward. One could therefore assume that flower mummification is not really senescence, in the genetic sense, but only dehydration.

In contrast to flower mummification, bent neck often leads to a relatively short VL. It is defined as a bending of the peduncle with an angle greater than 90°. It occurs due to either an air embolism or the accumulation of microorganisms at or within the stem end, which blocks the xylem vessels and prevents water uptake [100]. It is combined to low lignin and pectin biosynthesis in some rose cultivars and to the upregulation of genes related to starch breakdown, and to the upregulation of several NAC and WRKY TFs associated with dehydration-induced senescence [101, 102]. Although similar, this phenomenon is different from the one observed in living flowers, in which it is considered as a developmental process involving phenylpropanoids, auxin, CK, and GA [101, 103, 104].

The VL terminating symptom linked to *Botrytis cinerea* infection influences the aesthetic value of flowers. It is related to the susceptibility of the genotype to the pathogen, as well as other external factors such as growth conditions (production site location, humidity, etc.) and handling of the cut flowers (humid condition during transportation or storage). Once the infection of *B. cinerea* becomes visible on one petal, it spreads to other neighboring petals in a relatively short time, resulting in a short VL value. Therefore, it is important to note that short VL due to external factors, such as pathogens, is not the same as short VL due to other petal senescence phenomena, and thus careful interpretation needs to be done. Moreover, one variety of rose can show several VL terminating symptoms. Interestingly, nano-silver, by inhibiting ethylene action, decreases the expression of *Bcspl1 (SNOD-PROT-LIKE 1)*, which encodes a putative virulence factor of *B. cinerea* in cut roses [97, 105]. This could explain the link between rose genotype and *B. cinerea* infection that is sometimes observed.

In the literature, the distinction between studies on whole-plant senescence and cut flowers or petal fragments is not always clear. Numerous studies on the flower senescence of roses have been carried out using different systems. In some studies, flowers attached to the plant are used, including analysis on transformed plants, but in others, cut flowers, detached petals, or petal disks are fed by different solutions or treated in different conditions [23, 46, 63]. While interesting findings were obtained using these two approaches, no clear distinction has been made in interpreting the results and their effects on petal senescence. Jones [27] pointed out the importance to distinguish the employed methods, particularly when it comes to the study on the nutrient remobilization phase of senescence. This distinction can help provide a more comprehensive understanding of the processes involved in petal senescence. As such, the nutrient remobilization is reduced in detached flowers as compared to attached ones, although senescence phenotypes in both conditions are visually similar. It is therefore worth considering that mechanisms associated with petal senescence may somehow differ in attached flowers and cut flowers, especially if post-harvest procedures were used. To provide some answers to this open question, we set up an experiment where we compared the VL and the lifespan (LS)—the time for the flower to progress from blooming to death when attached to the plant—in five rose cultivars (Fig. 3). Our results show that VL and LS are identical, both in cultivars with a short VL (1283-18-03 and MEIzhoro) or with a long VL (MEIgriotti, MEIcrakaba, MEIrvingaye). Although this experiment does not reveal whether physiological processes occurring in both sets of flowers are identical, it suggests that senescence might be comparable in cut and attached flower. Indeed, Paramita [106] observed that the lifespan of a potted rose is not significantly different from that of a cut rose placed in a vase. This is equally true of varieties whose flowers faded quickly as of those whose flowers last much longer (Fig. 3).

## Conclusion and future prospects

The fact that gene expression and hormonal regulation might be different between cut flowers and flowers aging still attached to the plant seems like a reasonable assumption. Yet, current knowledge is insufficient to assess to what extent senescence relies on common physiological processes in each case. It seems that common points exist, e.g. regarding the induction of petal senescence by ethylene. Perhaps, the distinction is not so evident, given that some studies, most notably physiological ones, rely on flowers or parts of flowers detached from the plant, thus confusing the effects of both flower cutting and aging. Much work has sought to improve the VL of cut roses, often in an empirical manner. For example, environmental conditions and genotypes have been tested for better resistance of the flowers to pre-harvest treatments, dehydration, transportation, etc. [73, 88, 99, 102, 105]. Therefore, comprehensive work comparing the genome expression of cut and uncut flowers from several cultivars would be of great interest, to gain both new fundamental knowledge and understanding of petal senescence, and for the horticultural profession to help with the selection of long-VL varieties. The fact that VL and LS are widely identical in several cultivars should help in facilitating comparisons with such an approach, as it implies that aging progression, and by extension phenotypical consequences, are likely synchronized between both sets of flowers. Whether genetic determinants are identical in both types of senescence remains to be determined. On the whole, comparative studies focusing on genetic and environmental factors between cut roses and potted or garden roses would be invaluable for the cut flower industry.

## Supplementary Material

Web_Material_uhaf342
